# CF_3_-Substituted Mollugin 2-(4-Morpholinyl)-ethyl ester as a Potential Anti-inflammatory Agent with Improved Aqueous Solubility and Metabolic Stability

**DOI:** 10.3390/molecules23082030

**Published:** 2018-08-14

**Authors:** Ki Bum Hong, Darong Kim, Bo-Kyung Kim, Seo Yeon Woo, Ji Hoon Lee, Seung-Hee Han, Gyu-Un Bae, Soosung Kang

**Affiliations:** 1 New Drug Development Center, Daegu-Gyeongbuk Medical Innovation Foundation (DGMIF), Daegu 41061, Korea; kbhong@dgmif.re.kr (K.B.H.); kdrlj237@dgmif.re.kr (D.K.); kbky9872@dgmif.re.kr (B.-K.K.); sywoo@dgmif.re.kr (S.Y.W.); jhlee@dgmif.re.kr (J.H.L.); 2 Central Research Laboratory, KOREA PHARMA Co. Ltd., jeyakgongdan 3-gil, Hyangnam-eup, Hwaseong-si, Gyeonggi-do 18622, Korea; seunghee.han@koreapharma.co.kr; 3 Research Center for Cell Fate Control, College of Pharmacy, Sookmyung Women’s University, Seoul 03760, Korea; gbae@sm.ac.kr; 4 College of Pharmacy and Graduate School of Pharmaceutical Sciences, Ewha Womans University, Seoul 03760, Korea

**Keywords:** mollugin, inflammatory bowel disease, ADME, solubility

## Abstract

Although mollugin, the main ingredient of the oriental medicinal herb *Rubia cordifolia,* has considerable anti-inflammatory effects, it has poor aqueous solubility as well as poor metabolic and plasma stability. To overcome these shortfalls, various mollugin derivatives have been synthesized and evaluated for their ability to inhibit U937 monocyte cell adhesion to HT-29 colonic epithelial cells in TNF-α- or IL-6-induced models of colon inflammation. The 2-(4-morpholinyl)-ethyl ester of CF3-substituted mollugin (compound **15c**) showed good water solubility, improved metabolic and plasma stability, and greater inhibitory activity than mesalazine in both the TNF-α- and IL-6-induced colonic epithelial cell adhesion assays, suggesting that **15c** is a potential anti-inflammatory agent.

## 1. Introduction

**Figure 1:**
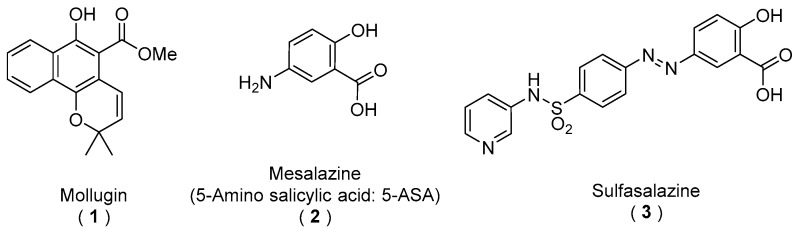
Structures of mollugin (**1**), mesalazine (**2**), and sulfasalazine (**3**).

*Rubia cordifolia* is a flowering plant in the coffee family (*Rubiaceae*), widely distributed from Africa to Asia and Australia [1]. Its roots have been used as a traditional medicine in India [2] and China [3] for their anti-inflammatory, astringent, tonic, antiseptic, deobstruent, and antidysenteric effects [4,5]. Mollugin (**1**), a methyl ester derivative of naphthoquinone (Figure 1), has been identified as a major active component of the roots of *Rubia cordifolia* [6]. Recent pharmacological data show that mollugin (**1**) manifests a wide range of biologically interesting properties, such as antibacterial, anti-inflammatory, antileukemia, and anti-allergic activities [7]. In particular, mollugin (**1**) displays efficacy against inflammatory bowel disease (IBD) in both a TNF-α-induced colon inflammation cell culture model and a DSS/TNBS-induced IBD animal model [8]. In the IBD study, mollugin inhibited NF-κB activation [9] and blocked the JAK-STAT signaling pathway [5]. As these results became well known, the naphthoquinone scaffold of mollugin was spotlighted. Diverse analogues [7,10] such as oxomollugin [11] and azamollugin [12] were synthesized to explore their anti-inflammatory, antioxidant, antibacterial, and anticancer activities.

Regarding IBD medicines on the market, only a few synthetic anti-inflammatory drugs, such as mesalazine (**2**) [13] and sulfasalazine (**3**) [14] (Figure 1), are used in mild to moderate cases. However, those 4-aminosalicylic acid-type drugs have several drawbacks, such as the need for high dosages (3 g/day), low efficacy, and various adverse effects [13]. Because alternative medications and treatments such as corticosteroid therapy or the immunosuppressive agent infliximab [15] are also of limited use due to various serious adverse effects and treatment costs, an urgent and medically unmet need exists for the development of new oral drugs with improved efficacy and lower toxicity in comparison with the current drug mesalazine. Because mollugin had at least 1000-fold improved efficacy over mesalazine in the cell adhesion assay and in an IBD animal model [8], our previous efforts focused on development of mollugin as a therapeutic agent for patients with IBD. However, further study with mollugin was limited due to uneven assay results caused by its poor aqueous solubility as well as metabolic instability.

As a part of our ongoing research on the development of IBD treatments using mollugin, we have modified the methyl ester of mollugin to various aminoalkyl esters to increase its water solubility [16] via formation of an ammonium salt. We also added F, CH_3_, and CF_3_ groups to the naphthalene ring of mollugin to increase its metabolic stability. Herein, we report the synthesis of various mollugin esters and substituted mollugins and their water solubility, anti-IBD effects in the colonic epithelial cell adhesion assay, metabolic stability, and plasma stability.

## 2. Results and Discussion

### 2.1. Chemistry

**Scheme 1:**
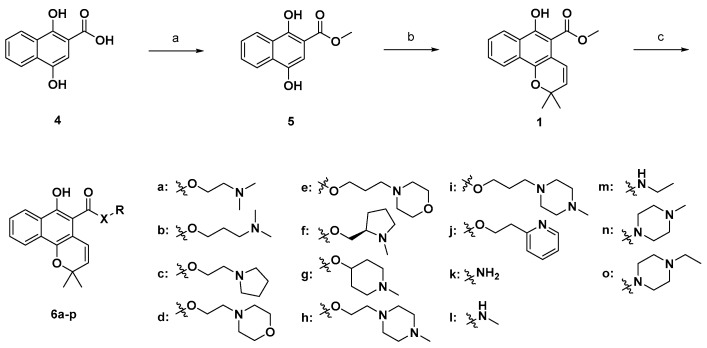
Synthesis of various mollugin derivatives. *Reagents and conditions*: (a) Me_2_SO_4_ (1.05 eq.), NaHCO_3_ (2.0 eq.), DMF, r.t., 5 h, 96%; (b) 3-methylbut-2-enal (2.2 eq.), PhB(OH)_2_ (1.1 eq.), AcOH, toluene, reflux, 20 h, 67%; (c) alcohol or amine TsOH (5%), microwave, 160 °C, 3 h, 8–52%.

The general procedure for the synthesis of mollugin derivatives **6a**–**o** is depicted in Scheme 1.

Mollugin (**1**) was prepared from commercially available 1,4-dihydroxy-2-naphthoic acid (**4**) by known methodology [17,18,19,20]. Microwave-assisted transesterification of methyl ester of mollugin with various alcohols and catalytic *p*-toluenesulfonic acid gave target products **6a**–**o**. Although the product yields at fixed reaction times in the microwave reactor were moderate to low, other side products were not formed and the mollugin starting material could be recovered after the reaction.

**Scheme 2:**
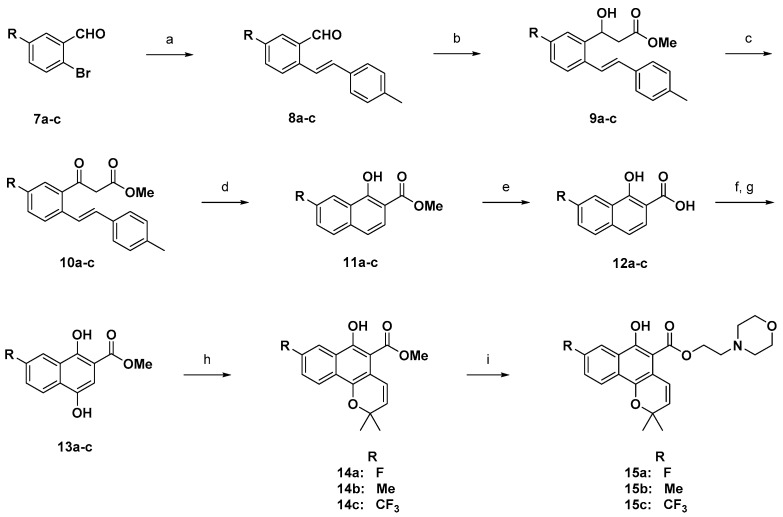
Synthesis of mollugin derivatives **14a**–**c** and **15a**–**c**. *Reagents and conditions*: (a) Pd(OAc)_2_ (0.1 eq.), Tri(*o*-tolyl)phosphine (0.2 eq.), TEA, 130 °C, 15 h, yield: 68–75%; (b) Zinc (1.5 eq.), Benzene, 110 °C, 5 h, yield: 79–82%; (c) Dess-Martin periodinane (1.3 eq.), DCM, r.t, 2 h, yield: 86–90%; (d) Pd(TFA)_2_ (0.2 eq.), Cu(OAc)_2_ (1.0 eq.), DCE, 100 °C, 10 h, yield: 90–95%; (e) aqueous KOH, THF, 95 °C, 20 h, yield: 95–98% (f) K_2_S_2_O_8_ (1.5 eq.) in water, 10% NaOH water solution, 1,4-dioxane, 0 °C to r.t., overnight; (g) NaHCO_3_ (3 eq.), iodomethane (3 eq.), DMF, 40 °C, overnight, yield: 80–82%; (h) PhB(OH)_2_ (2.0 eq.), AcOH (5.0 eq.), toluene, 130 °C, 6 h, yield: 63–66%; (i) 2-morpholinoethan-1-ol (10 eq.), CH_3_ONa (2.0 eq.), toluene, microwave (150 °C, 1 h), yield: 40–45%.

The synthetic procedure for mollugin derivatives **15a**–**c** is outlined in Scheme 2. Intermediates **8a**–**c**, obtained by the Wittig olefination between F-, CH_3_-, and CF_3_-substituted aldehydes and 4-methylstyrene, were treated with methyl 2-bromoacetate under zinc-mediated coupling conditions to yield alcohols **9a**–**c** at 79–82% yields. Diketones **10a**–**c**, which were prepared from DMP oxidation of secondary alcohol in **9a**–**c**, were subjected to a Pd-catalyzed cyclization reaction to yield naphthols **11a**–**c** at 90–95% yields. Hydrolysis and potassium persulfate-mediated oxidation and esterification yielded the mollugin core (1,4-dihydroxynaphthalene) **13a**–**c** at 80–82% yields. The cyclized adducts **14a**–**c** were obtained by a known method [17,18], and final direct esterification was performed by microwave irradiation to yield substituted mollugins **15a**–**c** at 40–45% yields.

**Scheme 3:**
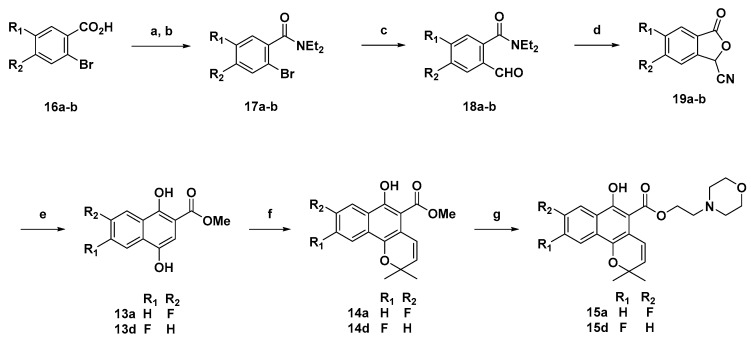
Alternative synthetic route for **15a** and **15d**. *Reagents and conditions*: (a) oxalyl chloride; (b) HN(Et)_2_; (c) BuLi, DMF; (d) 18-crown ether, KCN, TMSCN; (e) methyl acrylate, LiOtBu, THF, 8 h; (f) PhB(OH)_2_, AcOH, toluene, 130 °C, 6 h; (g) 2-morpholinoethan-1-ol, CH_3_ONa, toluene, microwave (150 °C, 1 h).

Alternatively, compounds **15a**,**d** were prepared from **16a**,**b** via Hauser annulation [21] (Scheme 3). The benzoic acids **16a**–**b** were converted to diethylamide **17a**–**b** via acyl halide formation, followed by reaction with diethylamine. Lithiation of **17a**–**b**, which was followed by quenching with anhydrous DMF, provided aldehyde **18a**–**b** in good yields. Subsequent treatment of formylbenzamide **18a**–**b** with trimethylsilyl cyanide in the presence of KCN and 18-crown-6 gave the corresponding key intermediates **19a**–**b** [22]. Hauser annulation of cyanophthalide **19a**–**b** with methyl acrylate and LiOtBu yielded the fluorinated 1,4-naphthoquinol **13a**,**d**, which further underwent cyclization and transesterification to yield **15a**,**d**.

### 2.2. Biological Activity

Synthesized mollugin derivatives were subjected to the colonic epithelial cell adhesion assay [8], which mimics the initial stage of colon inflammation. The inhibition of TNF-α-induced or IL-6-induced adhesion of U937 monocytic cells to HT-29 cells in 10 μM solution of synthesized molecules was measured, and the results are summarized in Table 1 and Table 2. Mesalazine (**2**), a commercially available medication for the treatment of IBD, and mollugin were chosen as assay positive controls. As shown in Table 1, mesalazine displayed ~30% inhibition of U937 adhesion to HT-29 cells at a 20 mM concentration. Surprisingly, at 2000-fold dilution (10 μM), monocyte cell adhesion to HT-29 was reduced to 40% when the cells were treated with mollugin or its various ester derivatives. In particular, **6a**–**b**, **6d**–**g**, **6i**–**k**, and **6n** significantly inhibited both TNF-α- and IL-6-induced monocyte-epithelial adhesion. Although mollugin *N*-ethyl-pyrrolidine (**6c**), *N*-ethyl-piperazine esters (**6h**), and various amides (**6l**–**m**, **6o**) showed good % inhibition in the TNF-α-induced adhesion model, they displayed reduced % inhibition in the IL-6-induced adhesion model.

Compounds **6a**–**j** and **6n**–**o** have a tertiary amine in their side chains; they showed good water solubility (>10 mM). Thus, the substitution of the methyl ester of mollugin with various solubilizing aminoalkyl esters enhances the aqueous solubility of the mollugin without significant reduction of inhibition capability in the TNF-α- or IL-6-induced monocyte-epithelial adhesion assay.

Substitution of the methyl ester of mollugin with various aminoalkyl esters also resulted in improved plasma stability [23]; although mollugin, a methyl ester, was unstable in rat plasma, aminoalkyl esters of mollugin **6d**–**h** displayed good stabilities in rat and human plasma. Among the water-soluble mollugin derivatives (**6a**–**i** and **6n**–**o**), compounds **6h** and **6i**, which have a piperazine moiety at the ester, were excluded because their IC_50_s for hERG channel inhibition [24] were 3.3 μM and 5.3 μM, respectively. They may therefore have cardiovascular adverse effects. However, it was shown that mollugin itself and its ester or amide derivatives (**6a**–**b**, **6d**–**h**, **6k**–**l)** were generally unstable, especially with dog, rat, and mouse liver microsomes in the metabolic stability assay [25]. On the basis of those initial in vitro ADME studies, the 2-(4-morpholinyl)-ethyl group of **6d** was chosen as the alternative to methyl ester in mollugin for water solubility and plasma stability, but **6d** still needs further modification to improve its metabolic stability, especially in small animals. 

To improve metabolic stability by blocking the metabolic hydroxylation of the aromatic ring, F-, CH_3_-, or CF_3_- moieties were added to the naphthalene ring of mollugin [26]. The bioactivity, metabolic stability, and plasma stability of the F-, CH_3_-, or CF_3_-substituted molecules **14a**–**d** and their 2-(4-morpholinyl)-ethyl esters **15a**–**d** are summarized in Table 2. The bioactivity levels of **14a**–**d** in the TNF-α- or IL-6-induced monocyte-epithelial adhesion assay were similar or slightly better than mollugin. The metabolic stability of those molecules with rat or mouse liver microsomes improved over that of mollugin. As it is known that stability in human plasma is usually greater than that in rodent plasma [27], **15a**–**d** are very stable in human plasma but displayed slightly reduced stability in rat plasma. It is noteworthy that the 2-(4-morpholinyl)-ethyl ester derivatives **15a**–**d** had good inhibitory activity in the IL-6-induced monocyte-epithelial adhesion assay. They displayed over 50% inhibition of U937 adhesion to HT-29 cells at a 10 μM concentration. Based on the in vitro data, compound **15c** was chosen as a lead, since it displayed better bioactivity than mollugin with favorable metabolic and plasma stability as well as good water solubility (>10 mM).

**Figure 2:**
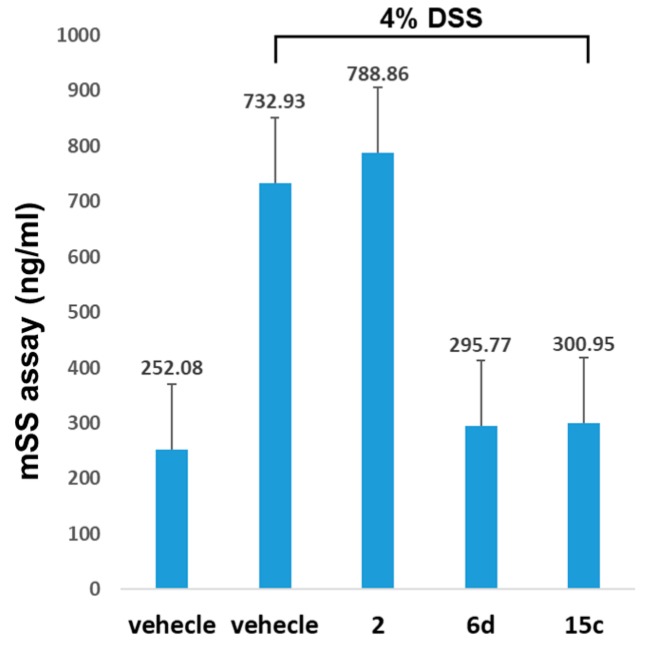
Analysis of mouse serum amyloid A (SAA) level. Effect of 6d on the level of SAA in the 4% dextran sulfate sodium (DSS)-induced mouse model. The level of SAA in serum was determined by ELISA. The control mice received water + vehicle (water), and the treated groups received 4% DSS + vehicle or DSS with mesalazine (20 mM), **6d** (10 μM), or **15c** (10 μM).

As shown in Table 3, the results of the CYP inhibition assay suggest that **15c** has no significant interaction (<50% inhibition at 10 μM drug concentration) with five major isozymes of CYPs (CYP 3A4, 2D6, 2C9, 1A2, 2C19). An MTT cell viability assay was performed, and the results suggested that neither **6d** nor **15c** affects the cell viability at 10 μM concentration. Serum amyloid A (SAA), one of the most well-known acute-phase proteins in mice [28], is up-regulated in the presence of DSS or DNBS stimulation of IBD and is measured for monitoring IBD [29]. We confirmed that **6d** or **15c** treatment reduces serum SAA level in a murine model of DSS-induced colitis (Figure 2).

## 3. Materials and Methods

### 3.1. Synthetic Methods and Molecular Characterization

All starting materials were purchased from Sigma-Aldrich (St. Louis, MO, USA), Alfa-Aesar (Ward Hill, MA, USA), and TCI (Nihonbashi, Japan) and were used without further purification. Reactions were performed under an atmosphere of dry nitrogen. An Initiator microwave system (Biotage, Uppsala, Sweden) was used for microwave-assisted reaction. LC-MS was performed on a system consisting of an electrospray ionization (ESI) source in a LCMS-2020 liquid chromatography-mass spectrometer system (Shimadzu, Kyoto, Japan; column: Shim-pack GIS, 100 × 3.0 mm, 3 μm ODS). A Teledyne ISCO flash purification system (Lincoln, NE, USA) with various prepacked silica gel cartridges was used for flash column chromatography. ^1^H- and ^13^C-NMR spectra were recorded in the indicated solvent on an AVANCE III HD (400 and 100 MHz for ^1^H and ^13^C, respectively) spectrometer (Bruker, Billerica, MA, USA). Chemical shifts are reported as δ values in parts per million downfield from TMS (δ 0.0) as the internal standard in CD_3_OD, DMSO-*d*_6_ or CDCl_3_. The purity of the compounds was evaluated on a Shimadzu reverse-phase analytical HPLC system (column: Ace C18, 150 × 4.6 mm, 3 μm). Purities of all compounds that were subjected to biological assay were >95%.

### 3.2. General Method A *(**6a**–**o**)*

The mixture of mollugin (0.35 mmol), alcohol (3.52 mmol), and catalytic *p*-TsOH (0.035 mmol) in 2 mL microwave vial was placed in the cavity of microwave reactor, and then stirred for 3 h at 160 °C. The produced brown mixture was dried under vacuum and subjected to purification (20 g silica gel cartridge, dichloromethane-MeOH) to give the title product.

*2-(Dimethylamino)ethyl 6-hydroxy-2,2-dimethyl-2H-benzo[h]chromene-5-carboxylate* (**6a**). Yield: 43%; yellow oil; ^1^H-NMR (CDCl_3_) *δ* 11.76 (bs, 1H), 8.36 (d, *J* = 8.4 Hz, 1H), 8.17 (d, *J* = 8.0 Hz, 1H), 7.61 (t, *J* = 7.2 Hz, 1H), 7.51 (t, *J* = 7.2 Hz, 1H), 7.10 (d, *J* = 10.0 Hz, 1H), 5.67 (d, *J* = 10.0 Hz, 1H), 4.54 (d, *J* = 6.0 Hz, 2H), 2.76 (d, *J* = 6.0 Hz, 2H), 2.35 (s, 6H), 1.48 (s, 6H); ^13^C-NMR (CDCl_3_) *δ* 170.42, 154.37, 141.43, 128.99, 128.89, 128.68, 126.12, 125.27, 123.98, 122.00, 121.84, 112.84, 103.81, 74.84, 62.66, 57.17, 45.43, 26.96; MS (ESI, *m*/*z*): calcd for C_20_H_24_NO_4_ [M + H]^+^ 342, found 342.

*3-(Dimethylamino)propyl 6-hydroxy-2,2-dimethyl-2H-benzo[h]chromene-5-carboxylate* (**6b**): Yield: 31%; yellow gel; ^1^H-NMR (CDCl_3_) *δ* 12.18 (bs, 1H), 8.36 (dd, *J* = 8.3, 0.4 Hz, 1H), 8.17 (d, *J* = 8.0 Hz, 1H), 7.62–7.59 (m, 1H), 7.52–7.48 (m, 1H), 7.13 (d, *J* = 10.0 Hz, 1H), 5.66 (d, *J* = 10.0 Hz, 1H), 4.49 (dd, *J* = 6.4 Hz, 2H), 2.47 (dd, *J* = 7.1 Hz, 2H), 2.28 (s, 6H), 2.04–1.97 (m, 2H), 1.49 (s, 6H); MS (ESI, *m*/*z*) calcd for C_21_H_26_NO_4_ [M + H]^+^ 356, found 356.

*2-(Pyrrolidin-1-yl)ethyl 6-hydroxy-2,2-dimethyl-2H-benzo[h]chromene-5-carboxylate* (**6c**): Yield: 9%; brown oil; ^1^H-NMR (CDCl_3_) *δ* 11.81 (bs, 1H), 8.35 (d, *J* = 8.3 Hz, 1H), 8.16 (d, *J* = 8.1 Hz, 1H), 7.60–7.56 (m, 1H), 7.52–7.47 (m, 1H), 7.09 (d, *J* = 10.0 Hz, 1H), 5.66 (d, *J* = 10.0 Hz, 1H), 4.57 (t, *J* = 5.9 Hz, 2H), 2.91 (t, *J* = 5.9 Hz, 2H), 2.66–7.64 (m, 4H), 1.85–1.82 (m, 4H), 1.49 (s, 6H); MS (ESI, *m*/*z*) calcd for C_22_H_26_NO_4_ [M + H]^+^ 368, found 368.

*2-Morpholinoethyl 6-hydroxy-2,2-dimethyl-2H-benzo[h]chromene-5-carboxylate* (**6d**): Yield: 30%; yellow gel; ^1^H-NMR (CDCl_3_) *δ* 11.61 (s, 1H), 8.36 (d, *J* = 8.0 Hz, 1H), 8.17 (d, *J* = 8.0 Hz, 1H), 7.62–7.58 (m, 1H), 7.52–7.48 (m, 1H), 7.18 (d, *J* = 10.0 Hz, 1H), 5.66 (d, *J* = 10.0 Hz, 1H), 4.55 (t, *J* = 5.6 Hz, 2H), 3.76 (t, *J* = 4.6 Hz, 4H), 2.80 (t, *J* = 5.7 Hz, 2H), 2.59 (t, *J* = 4.3 Hz, 4H), 1.49 (s, 6H); ^13^C-NMR (DMSO-*d_6_*) *δ* 168.3, 149.8, 140.8, 129.9, 128.7, 127.1, 126.5, 124.9, 123.4, 121.4, 121.1, 112.4, 107.2, 75.1, 63.1, 59.9, 54.2, 51.5, 26.5; MS (ESI, *m*/*z*) calcd for C_22_H_26_NO_5_ [M + H]^+^ 384, found 384.

*3-Morpholinopropyl 6-hydroxy-2,2-dimethyl-2H-benzo[h]chromene-5-carboxylate* (**6e**): Yield: 23%; yellow gel; ^1^H-NMR (CDCl_3_) *δ* 11.99 (bs, 1H), 8.36 (d, *J* = 8.3 Hz, 1H), 8.18 (d, *J* = 8.3 Hz, 1H), 7.65–7.61 (m, 1H), 7.55–7.51 (m, 1H), 6.99 (d, *J* = 10.0 Hz, 1H), 5.69 (d, *J* = 10.0 Hz, 1H), 4.53 (t, *J* = 6.1 Hz, 2H), 3.99 (t, *J* = 4.7 Hz, 4H), 3.17–3.13 (m, 4H), 2.37–2.30 (m, 2H), 1.85–1.60 (m, 2H), 1.50 (s, 6H); MS (ESI, *m*/*z*) calcd for C_23_H_28_NO_5_ [M + H]^+^ 398, found 398.

*(R)-(1-Methylpyrrolidin-2-yl)methyl 6-hydroxy-2,2-dimethyl-2H-benzo[h]chromene-5-carboxylate* (**6f**): Yield: 16%; yellow oil; ^1^H-NMR (CDCl_3_) *δ* 11.92 (s, 1H), 8.35 (d, *J* = 8.3 Hz, 1H), 8.16 (d, *J* = 8.3 Hz, 1H), 7.62–7.57 (m, 1H), 7.51–7.47 (m, 1H), 7.17 (d, *J* = 10.0 Hz, 1H), 5.67 (d, *J* = 10.0 Hz, 1H), 5.28–5.24 (m, 1H), 3.67 (d, *J* = 6.0 Hz, 2H), 2.80 (m, 1H), 2.51 (m, 2H), 2.37–2.32 (m, 4H), 2.00–1.90 (m, 3H), 1.50 (s, 6H); MS (ESI, *m*/*z*) calcd for C_22_H_26_NO_4_ [M + H]^+^ 368, found 368.

*1-Methylpiperidin-4-yl 6-hydroxy-2,2-dimethyl-2H-benzo[h]chromene-5-carboxylate* (**6g**): Yield: 17%; pale green oil; ^1^H-NMR (CDCl_3_) *δ* 11.97 (s, 1H), 8.36 (d, *J* = 8.2 Hz, 1H), 8.17 (d, *J* = 8.3 Hz, 1H), 7.63–7.59 (m, 1H), 7.53–7.49 (m, 1H), 7.23 (d, *J* = 10.0 Hz, 1H), 5.66 (d, *J* = 10.0 Hz, 1H), 4.59 (m, 2H), 3.89 (m, 2H), 3.72 (m, 2H), 3.59 (m, 2H), 3.41 (s, 3H), 3.38 (m, 1H), 1.49 (s, 6H); MS (ESI, *m*/*z*) calcd for C_22_H_26_NO_4_ [M + H]^+^ 368, found 368.

*2-(4-Methylpiperazin-1-yl)ethyl 6-hydroxy-2,2-dimethyl-2H-benzo[h]chromene-5-carboxylate* (**6h**): Yield: 34%; brown oil; ^1^H-NMR (MeOD) *δ* 8.36 (d, *J* = 8.2 Hz, 1H), 8.17 (d, *J* = 8.0 Hz, 1H), 7.62–7.58 (m, 1H), 7.52–7.48 (m, 1H), 7.16 (d, *J* = 10.0 Hz, 1H), 5.66 (d, *J* = 10.0 Hz, 1H), 4.55 (t, *J* = 5.6 Hz, 2H), 2.80 (t, *J* = 5.7 Hz, 2H), 2.63–2.47 (m, 8H), 2.31 (s, 3H), 1.49 (s, 6H); MS (ESI, *m*/*z*) calcd for C_23_H_29_N_2_O_4_ [M + H]^+^ 397, found 397.

*3-(4-Methylpiperazin-1-yl)propyl 6-hydroxy-2,2-dimethyl-2H-benzo[h]chromene-5-carboxylate* (**6i**): Yield: 13%; yellow gel; ^1^H-NMR (MeOD) *δ* 8.34 (d, *J* = 8.3 Hz, 1H), 8.16 (d, *J* = 8.2 Hz, 1H), 7.66 (m, 1H), 7.56 (m, 1H), 7.14 (d, *J* = 10.0 Hz, 1H), 5.83 (d, *J* = 10.0 Hz, 1H), 4.61 (t, *J* = 6.2 Hz, 2H), 3.76 (m, 2H), 3.37 (s, 8H), 3.02 (s, 3H), 2.36 (m, 2H), 1.51 (s, 6H); MS (ESI, *m*/*z*) calcd for C_24_H_31_N_2_O_4_ [M + H]^+^ 411, found 411.

*2-(Pyridin-2-yl)ethyl 6-hydroxy-2,2-dimethyl-2H-benzo[h]chromene-5-carboxylate* (**6j**): Yield: 11%; yellow oil; ^1^H-NMR (CDCl_3_) *δ* 12.00 (bs, 1H), 8.95 (d, *J* = 5.3 Hz, 1H), 8.33 (d, *J* = 8.3 Hz, 1H), 8.22 (m, 1H), 8.15 (d, *J* = 8.3 Hz, 1H), 7.78–7.72 (m, 2H), 7.64–7.60 (m, 1H), 7.53–7.50 (m, 1H), 6.78 (d, *J* = 10.0 Hz, 1H), 5.56 (d, *J* = 10.0 Hz, 1H), 4.88 (d, *J* = 6.0 Hz, 2H), 3.67 (d, *J* = 6.0 Hz, 2H), 1.46 (s, 6H); MS (ESI, *m*/*z*) calcd for C_23_H_22_NO_4_ [M + H]^+^ 376, found 376.

*6-Hydroxy-2,2-dimethyl-2H-benzo[h]chromene-5-carboxamide* (**6k**): Yield: 35%; brown gel; ^1^H-NMR (CDCl_3_) *δ* 12.00 (s, 1H), 8.39 (d, *J* = 8.2 Hz, 1H), 8.20 (d, *J* = 8.2 Hz, 1H), 7.67–7.64 (m, 1H), 7.56–7.52 (m, 1H), 7.35–7.26 (m, 3H), 5.73 (d, *J* = 9.9 Hz, 1H), 1.51 (s, 6H); MS (ESI, *m*/*z*) calcd for C_16_H_16_NO_3_ [M + H]^+^ 270, found 270.

*6-Hydroxy-N,2,2-trimethyl-2H-benzo[h]chromene-5-carboxamide* (**6l**): Yield: 52%; yellow solid; HPLC purity: 4.5 min, 96.3%; ^1^H-NMR (CDCl_3_) *δ* 12.23 (s, 1H), 8.34–8.32 (m, 1H), 8.17–8.14 (m, 1H), 7.60–7.55 (m, 1H), 7.53–7.49 (m, 1H), 6.68 (d, *J* = 10.0 Hz, 1H), 5.90 (s, 1H), 5.74 (d, *J* = 10.0 Hz, 1H), 3.08 (d, *J* = 4.9 Hz, 3H), 1.53 (s, 6H); MS (ESI, *m*/*z*) calcd for C_17_H_18_NO_3_ [M + H]^+^ 284, found 284.

*N-Ethyl-6-hydroxy-2,2-dimethyl-2H-benzo[h]chromene-5-carboxamide* (**6m**): Yield: 34%; orange solid; ^1^H-NMR (CDCl_3_) *δ* 12.20 (s, 1H), 8.32 (d, *J* = 8.0 Hz, 1H), 8.15 (d, *J* = 8.2 Hz, 1H), 7.59–7.55 (m, 1H), 7.53–7.49 (m, 1H), 6.69 (d, *J* = 9.7 Hz, 1H), 5.90 (m, 1H), 5.75 (d, *J* = 9.7 Hz, 1H), 3.60–3.53 (m, 2H), 1.54 (s, 6H), 1.30 (t, *J* = 7.2 Hz, 3H); MS (ESI, *m*/*z*) calcd for C_18_H_20_NO_3_ [M + H]^+^ 298, found 298.

*(6-Hydroxy-2,2-dimethyl-2H-benzo[h]chromen-5-yl)(4-methylpiperazin-1-yl)methanone* (**6n**): Yield: 32%; brown oil; ^1^H-NMR (CDCl_3_) *δ* 8.19 (d, *J* = 8.4 Hz, 1H), 8.09 (d, *J* = 8.4 Hz, 1H), 7.51 (m, 2H), 6.37 (d, *J* = 9.8 Hz, 1H), 5.69 (d, *J* = 9.8 Hz, 1H), 3.72 (m, 2H), 3.69 (bs, 1H), 3.61 (m, 2H), 2.53 (m, 2H), 2.33 (m, 2H), 2.30 (s, 3H), 1.49 (s, 6H); ^13^C-NMR (CDCl_3_) *δ* 169.02, 146.63, 141.53, 129.99, 127.28, 126.55, 126.14, 125.69, 122.97, 121.84, 120.63, 111.71, 110.64, 77.24, 75.61, 54.97, 45.89, 27.19 ; MS (ESI, *m*/*z*): calcd for C_21_H_25_N_2_O_3_ [M + H]^+^ 353, found 353.

*4-Ethylpiperazin-1-yl)(6-hydroxy-2,2-dimethyl-2H-benzo[h]chromen-5-yl)methanone* (**6o**): Yield: 16%; pale yellow gel; ^1^H-NMR (CDCl_3_) *δ* 12.20 (s, 1H), 8.22 (d, *J* = 7.8 Hz, 1H), 8.14 (d, *J* = 7.8 Hz, 1H), 7.51 (m, 2H), 6.38 (d, *J* = 9.8 Hz, 1H), 5.69 (d, *J* = 9.8 Hz, 1H), 3.76 (m, 2H), 3.51 (m, 2H), 2.58 (m, 2H), 2.30 (m, 2H), 2.42 (q, *J* = 7.2 Hz, 2H), 1.50 (s, 6H), 1.08 (t, *J* = 7.2 Hz, 3H); MS (ESI, *m*/*z*): calcd for C_22_H_27_N_2_O_3_ [M + H]^+^ 367, found 368.

### 3.3. General Method B *(*Compounds ***8a**–**c**)*

Various 2-bormobenzaldehyde (25 mmol), 4-methylstyrene (1.5 eq.), palladium (II) acetate (0.1 eq.) and tris(*o*-tolyl)phosphine (0.2 eq.) were dissolved in triethylamine (0.8 M) and added in a pressure tube. The pressure tube was tightly capped and heated for 15 h at 130 °C. The reaction mixture was quenched with saturated aqueous ammonium chloride and then organic materials were extracted with dichloromethane (two times). The combined organic layer was dried over magnesium sulfate, filtered, and concentrated under vacuum. The produced mixture was subjected to flash chromatography on silica gel using hexane/ethyl acetate (0 to 2% for 60 min) to give the wanted products. Yield 65–75%.

*(E)-5-Fluoro-2-(4-methylstyryl)benzaldehyde* (**8a**): Yield: 71%; Yellow oil; ^1^H-NMR (CDCl_3_) δ 10.32 (s, 1H), 7.82 (d, *J* = 16.1 Hz, 1H), 7.65–7.70 (m, 1H), 7.50–7.85 (m, 1H), 7.42–7.48 (m, 2H), 7.28–7.35 (m, 1H), 7.16–7.22 (m, 2H), 6.95 (d, *J* = 16.1 Hz, 1H), 2.38 (s, 3H); MS (ESI, *m*/*z*): calcd for C_16_H_14_FO [M + H]^+^ 241, found 241.

*(E)-5-Methyl-2-(4-methylstyryl)benzaldehyde* (**8b**): Yield: 73%; Yellow oil; ^1^H-NMR (CDCl_3_) δ 10.31 (s, 1H), 7.94 (d, *J* = 16.1 Hz, 1H), 7.58–7.65 (m, 2H), 7.42–7.49 (m, 2H), 7.35–7.41 (m, 1H), 7.18 (m, *J* = 7.8 Hz, 2H), 6.99 (d, *J* = 16.1 Hz, 1H), 2.42 (s, 3H), 2.37 (s, 3H); MS (ESI, *m*/*z*): calcd for C_17_H_17_O [M + H]^+^ 237, found 237. 

*(E)-5-Trifluoromethyl-2-(4-methylstyryl)benzaldehyde* (**8c**): Yield: 75%; Yellow oil; ^1^H-NMR (CDCl_3_) δ 10.32 (s, 1H), 7.81 (d, *J* = 16.1 Hz, 1H), 7.62–7.68 (m, 1H), 7.51–7.55 (m, 1H), 7.42–7.45 (m, 2H), 7.26–7.31 (m, 1H), 7.13–7.24 (m, 2H), 6.97 (d, *J* = 16.1 Hz, 1H), 2.38 (s, 3H); MS (ESI, *m*/*z*): calcd for C_17_H_14_F_3_O [M + H]^+^ 291, found 291.

### 3.4. General Method C *(*Compounds ***9a**–**c**)*

(4-Methylstyryl)benzaldehyde **8a**–**c** (15 mmol) was dissolved in anhydrous benzene (50 mL). To this solution, Zinc powder (1.5 eq.) and methyl 2-bromoacetate (2.0 eq.) were added to the reaction mixture, which was then refluxed for 5 h. The reaction was quenched with saturated aqueous ammonium chloride and the organic materials were extracted with dichloromethane (two times). The combined organic layer was dried over magnesium sulfate, filtered, and concentrated under vacuum. The mixture was purified by flash chromatography on silica gel using hexane/ethyl acetate (5 to 20% for 60 min) to give the target products; Yield 79–82%.

*Methyl (E)-3-hydroxy-3-(5-fluoro-2-(4-methylstyryl)phenyl)propionate* (**9a**): Yield: 79%; yellow oil; ^1^H-NMR (CDCl_3_) *δ* 7.50 (dd, *J* = 8.7, 5.75 Hz, 1H), 7.36–7.47 (m, 2H), 7.30 (dd, *J* = 10.0, 2.7 Hz, 1H), 7.12 (d, *J* = 16.1 Hz, 1H), 6.98 (td, *J* = 8.4, 2.81 Hz, 1H), 6.78–6.94 (m, 3H), 5.50 (dt, *J* = 9.2, 3.1 Hz, 1H), 3.83 (s, 3H), 3.73 (s, 3H), 3.37 (d, *J* = 3.2 Hz, 1H), 2.55–2.77 (m, 2H); MS (ESI, *m*/*z*): calcd for C_19_H_20_FO_3_ [M + H]^+^ 315, found 315.

*Methyl (E)-3-hydroxy-3-(5-methyl-2-(4-methylstyryl)phenyl)propanoate* (**9b**): Yield: 80%; yellow oil; ^1^H-NMR (CDCl_3_) *δ* 7.47 (d, *J* = 7.8 Hz, 1H); 7.36–7.43 (m, 3H), 7.32 (d, *J* = 15.9 Hz, 1H), 7.17 (d, *J* = 7.8 Hz, 2H), 7.08–7.13 (m, 1H), 6.91 (d, *J* = 15.9 Hz, 1H), 5.52 (ddd, *J* = 7.8, 4.9, 3.2 Hz, 1H), 3.72 (s, 3H), 3.21 (d, *J* = 2.9 Hz, 1H), 2.67–2.74 (m, 2H), 2.36 (ss, 6H); MS (ESI, *m*/*z*): calcd for C_20_H_23_O_3_ [M + H]^+^ 311, found 311.

*Methyl (E)-3-hydroxy-3-(5-(trifluoromethyl)-2-(4-methylstyryl)phenyl)propanoate* (**9c**): Yield: 82%; yellow oil; ^1^H-NMR (CDCl_3_) δ 7.86 (s, 1H) 7.66 (d, *J* = 8.3 Hz, 1H), 7.51–7.59 (m, 1H), 7.42 (m, *J* = 8.1 Hz, 2H), 7.30 (d, *J* = 15.9 Hz, 1H), 7.19 (m, *J* = 7.8 Hz, 2H), 7.02 (d, *J* = 15.9 Hz, 1H), 5.56 (dt, *J* = 8.1, 3.9 Hz, 1H), 3.73 (s, 3H), 3.47 (d, *J* = 3.2 Hz, 1H), 2.66–2.73 (m, 2H), 2.37 (s, 3H); MS (ESI, *m*/*z*): calcd for C_20_H_20_F_3_O_3_ [M + H]^+^ 365, found 365.

### 3.5. General Method D *(*Compounds ***10a**–**c**)*

Various methyl (4-methylstyryl) phenyl propanoate **9a**–**c** (12 mmol) and Dess-Martin periodinane (1.3 equiv) were dissolved in anhydrous dichloromethane, and stirred at room temperature for 2 h. The reaction mixture was quenched with saturated sodium bicarbonate water solution and extracted with dichloromethane (two times). The combined organic layer was washed with brine and dried over magnesium sulfate, filtered and concentrated under vacuum. The product was purified by flash chromatography on silica gel using hexane/ethyl acetate (1 to 5% for 60 min). Product obtained as a pale yellow oil. Yield 86–90%.

*Methyl (E)-3-(5-Fluoro-2-(4-methylstyryl)phenyl)-3-oxopropanoate* (**10a**): Yield: 87%; yellow oil; ^1^H-NMR (CDCl_3_) *δ* 7.61–7.71 (m, 1H), 7.50 (d, *J* = 16.4 Hz, 1H), 7.40 (t, *J* = 8.4 Hz, 2H), 7.28–7.36 (m, 1H), 7.09–7.24 (m, 3H), 6.85–6.98 (m, 1H), 3.94 (s, 2H), 3.77 (m, 3H), 2.36 (s, 3H); MS (ESI, *m*/*z*): calcd for C_19_H_18_FO_3_ [M + H]^+^ 313, found 313.

*Methyl (E)-3-(5-methyl-2-(4-methylstyryl)phenyl)-3-oxopropanoate* (**10b**): Yield: 86%; yellow oil; ^1^H-NMR (CDCl_3_) *δ* 7.50–7.67 (m, 2H), 7.27–7.44 (m, 4H), 7.16 (d, *J* = 8.1 Hz, 2H), 6.83–7.05 (m, 1H), 3.97 (s, 2H), 3.73–3.80 (m, 3H), 2.40 (s, 3 H), 2.35 (s, 3 H); MS (ESI, *m*/*z*): calcd for C_20_H_21_O_3_ [M + H]^+^ 309, found 309.

*Methyl (E)-3-(5-(trifluoromethyl)-2-(4-methylstyryl)phenyl)-3-oxopropanoate* (**10c**): Yield: 90%; yellow oil; ^1^H-NMR (CDCl_3_) *δ* 7.70–7.89 (m, 2H), 7.66 (dd, *J* = 8.3, 2.0 Hz, 1H), 7.57 (d, *J* = 16.1 Hz, 1H), 7.35–7.48 (m, 2H), 7.19 (d, *J* = 7.8 Hz, 2H), 7.08 (dd, *J* = 16.1, 13. 5 Hz, 1H), 3.99 (s, 2H), 3.74–3.82 (m, 3H), 2.38 (s, 3H); MS (ESI, *m*/*z*): calcd for C_20_H_18_F_3_O_3_ [M + H]^+^ 363, found 363.

### 3.6. General Method E *(*Compounds ***11a**–**c**)*

Various methyl (4-methylstyryl) phenyl) oxopropanoate **10a**–**c** (10 mmol) was dissolved in anhydrous dichloroethane (50 mL). Palladium (II) trifluoroacetate (0.2 equiv), copper (II) acetate (1.0 equiv) and methyl acrylate (3.0 equiv) were added in reaction mixture and stirred at 100 °C for 10 h. The reaction mixture was quenched with saturated ammonium chloride water solution and extracted with dichloromethane (two times). The combined organic layer was washed with brine and dried over magnesium sulfate, filtered and concentrated under vacuum. The product was purified by flash chromatography on silica gel using hexane/ethyl acetate (0 to 2% for 60 min). Product obtained as a white solid. Yield 90–95%.

*Methyl 7-fluoro-1-hydroxy-2-naphthoate* (**11a**): Yield: 90%; white solid; ^1^H-NMR (CDCl_3_) δ 11.91 (s, 1H), 8.01 (dd, *J* = 10.0, 2.7 Hz, 1H), 7.65–7.83 (m, 2H) 7.37 (td, *J* = 8.7, 2.7 Hz, 1H), 7.28 (d, *J* = 8.8 Hz, 1H), 4.01 (s, 3H); MS (ESI, *m*/*z*): calcd for C_12_H_10_FO_3_ [M + H]^+^ 221, found 221.

*Methyl 7-methyl-1-hydroxy-2-naphthoate* (**11b**): Yield: 92%; white solid; ^1^H-NMR (CDCl_3_) δ 11.95 (s, 1H), 8.19 (s, 1H), 7.67 (d, *J* = 8.3 Hz, 1H), 7.70 (d, *J* = 8.8 Hz, 1H), 7.44 (dd, *J* = 8.3, 1.7 Hz, 1H), 7.24 (d, *J* = 9.1 Hz, 1H), 4.00 (s, 3H), 2.54 (s, 3H); MS (ESI, *m*/*z*): calcd for C_13_H_13_O_3_ [M + H]^+^ 217, found 217.

*Methyl 7-(trifluoromethyl)-1-hydroxy-2-naphthoate* (**11c**): Yield: 95%; white solid; ^1^H-NMR (CDCl_3_) δ 12.07 (s, 1H), 8.72 (s, 1H), 7.82–7.95 (m, 2H), 7.76 (dd, *J* = 8. 6, 1.7 Hz, 1H), 7.33 (d, *J* = 8.8 Hz, 1H), 4.02 (s, 3 H); MS (ESI, *m*/*z*): calcd for C_13_H_9_F_3_O_3_ [M + H]^+^ 271, found 271.

### 3.7. General Method F *(*Compounds ***12a**–**c**)*

Various methyl hydroxy-2-naphthoate **11a**–**c** (9 mmol) was dissolved in tetrahydrofuran and stirred at 25 °C for 10 min. The excess amount of potassium hydroxide solution was added in the reaction mixture and stirred at 90 °C for 20 h. The reaction mixture was cooled, acidified with 6N hydrogen chloride solution and extracted with ethyl acetate (two times). The combined organic layer was washed with brine and dried over magnesium sulfate, filtered and concentrated under vacuum. The product was purified by flash chromatography on silica gel using dichloromethane/methanol (10 to 30% for 60 min). Product obtained as a yellow solid. Yield 95–98%.

*7-Fluoro-1-hydroxy-2-naphthoic acid* (**12a**): Yield: 95%; yellow solid; ^1^H-NMR (CD_3_OD) δ 8.14 (s, 1H), 7.76 (d, *J* = 8.7, 1H), 7.71 (d, *J* = 8.4, 1H), 7.45 (dd, *J* = 8.4, 1.8 Hz, 1H), 7.25 (d, *J* = 8.7, 1H); MS (ESI, *m*/*z*): calcd for C_11_H_8_FO_3_ [M + H]^+^ 207, found 207.

*7-Methyl-1-hydroxy-2-naphthoic acid* (**12b**): Yield: 97%; yellow solid; ^1^H-NMR (CD_3_OD) δ 7.94 (dd, *J* = 10.3, 2.7 Hz, 1H), 7.89–7.81 (m, 2H), 7.40 (td, *J* = 8.7, 2.7 Hz, 1H), 7.32 (d, *J* = 8.7, 1H); MS (ESI, *m*/*z*): calcd for C_12_H_11_O_3_ [M + H]^+^ 203, found 203.

*7-(Trifluoromethyl)-1-hydroxy-2-naphthoic acid* (**12c**): Yield: 98%; yellow solid; ^1^H-NMR (MeOD) δ 7.91 (dd, *J* = 10.3, 2. 5 Hz, 1H), 7.87–7.75 (m, 2H), 7.38 (td, *J* = 8.7, 2.7 Hz, 1H), 7.33–7.24 (m, 1 H); MS (ESI, *m*/*z*): calcd for C_12_H_8_F_3_O_3_ [M + H]^+^ 257, found 257.

### 3.8. General Method G *(*Compounds ***13a**–**c**)*

Various 1-hydroxy-2-naphthoic acids **12a**–**c** (6 mmol) was dissolved in a mixed solution of 10% sodium hydroxide and 1,4-dioxane and stirred at 0 °C for 1 h. A saturated aqueous solution of potassium persulfate (1.5 equiv) was slowly added to the reaction mixture during 4–5 h. The reaction mixture was continuously stirred at 20 °C for overnight. The reaction mixture was acidified with 6N HCl solution and extracted with ethyl acetate (two times). The combined organic layer was washed with brine and dried over magnesium sulfate, filtered and concentrated under vacuum. The dried mixture was dissolved in *N*,*N*-dimethyl-formamide, 3.0 equivalent of potassium hydrogen carbonate was added to reaction mixture. Which was stirred at 40 °C for 0.5 h 3.0 equivalent of Iodomethane was added in reaction mixture, which was stirred at 40 °C for overnight. The reaction mixture was quenched with saturated sodium bicarbonate water solution and extracted with dichloromethane (two times). The combined organic layer was washed with brine and dried over magnesium sulfate, filtered and concentrated under vacuum. The product was purified by flash chromatography on silica gel using hexane/ethyl acetate (5 to 10% for 60 min). Product obtained as a pale yellow solid. Yield 80–82%.

*Methyl 7-fluoro-1,4-dihydroxy-2-naphthoate* (**13a**): Yield: 81%; yellow solid; ^1^H-NMR (CDCl_3_) δ 9.80 (s, 1H), 8.08 (d, *J* = 8.0 Hz, 1H), 7.56 (d, *J* = 4.4 Hz, 2H), 7.40 (m, 1H), 7.14 (s, 1H), 3.96 (s, 3H); ^13^C-NMR (100 MHz, CDCl_3_) δ 170.5, 158.8, 152.0, 145.3, 127.9, 127.3, 120.4, 119.9, 119.0, 115.0, 106.4, 53.2; MS (ESI, *m*/*z*): calcd for C_12_H_10_FO_4_ [M + H]^+^ 237, found 237.

*Methyl 7-methyl-1,4-dihydroxy-2-naphthoate* (**13b**): Yield: 80%; yellow solid; ^1^H-NMR (CDCl_3_) δ 11.54 (s, 1H), 8.18 (s, 1H), 8.02 (d, *J* = 8. 6 Hz, 1H), 7.49 (dd, *J* = 8.4, 1.59 Hz, 1H), 7.04 (s, 1H), 4.84 (s, 1H), 3.98 (s, 3H), 2.55 (s, 3H); MS (ESI, *m*/*z*): calcd for C_13_H_13_O_4_ [M + H]^+^ 233, found 233.

*Methyl 7-(trifluoromethyl)-1,4-dihydroxy-2-2-naphthoate* (**13c**): Yield: 82%; yellow solid; ^1^H-NMR (CDCl_3_) δ 11.60 (s, 1H), 8.70 (s, 1H), 8.26 (d, *J* = 8. 6 Hz, 1H), 7.72–7.84 (d, *J* = 8. 6 Hz, 1H), 7.22 (s, 1H), 5.24 (s, 1H), 4.00 (s, 3H); MS (ESI, *m*/*z)*: calcd for C_13_H_9_F_3_O_4_ [M + H]^+^ 286, found 286.

### 3.9. Methyl fluoro-1,4-dihydroxy-2-naphthoates ***13a***,***d*** from ***19a**–**b***

LiOtBu (6 mL. 1 M in THF, 6.0 mmol) was added to stirred solution of the phthalide (0.35 g, 2.0 mmol) in dry THF (10 mL) at −60 °C under an inert atmosphere. The resulting solution was stirred at the same temperature for 30 min after which a solution of methyl acrylate (0.362 mL, 4.0 mmol) in dry THF (10 mL) was added. The reaction mixture was stirred for another 30 min at −60 °C followed by 8 h stirring at room temperature. The reaction was then quenched with saturated ammonium chloride solution (20 mL) and THF was evaporated under reduced pressure. The residue was then extracted with ethyl acetate (2 × 20 mL). The combined extracts were washed with brine, dried (Na_2_SO_4_), filtered, and concentrated to afford the crude product which was purified by column chromatography to obtain the pure compound. 

Methyl 7-fluoro-1,4-dihydroxy-2-naphthoate (**13a**): Yield = 95%. 

*Methyl 6-fluoro-1,4-dihydroxy-2-naphthoate* (**13d**): Yield = 56%; ^1^H-NMR (CDCl_3_) δ 11.59 (s, 1H), 8.40 (dd, *J* = 9.2, 5.7 Hz, 1H), 7.31 (dd, *J* = 10.2, 2.6 Hz, 1H), 7.31 (td, *J* = 8.7, 2.6 Hz, 1H), 7.13 (s, 1H), 4.97 (s, 1H), 3.98 (s, 3H); MS (ESI, *m*/*z*): calcd for C_12_H_10_FO_4_ [M + H]^+^ 237, found 237.

### 3.10. General Method H *(*Compounds ***14a**–**d**)*

Various methyl 1,4-dihydroxy-2-naphthoate derivatives **13a**–**d** (5 mmol), phenylboronic acid (2.0 equiv), glacial acetic acid (5.0 equiv), and 3-methylbut-2-enal (3.0 equiv) were dissolved in anhydrous toluene and refluxed for 6 h under nitrogen gas in an apparatus fitted with a Dean–Stark trap. The reaction mixture was cooled, quenched with saturated sodium bicarbonate water solution, and extracted with dichloromethane (two times). The combined organic layer was washed with brine and dried over magnesium sulfate, filtered and concentrated under vacuum. The product was purified by flash chromatography on silica gel using hexane/ethyl acetate (5 to 20% for 60 min). Product obtained as a pale yellow solid. Yield 63–66%;

*Methyl 8-fluoro-6-hydroxy-2,2-dimethyl-2H-benzo[h]chromene-5-carboxylate* (**14a**): Yield: 68%; yellow solid; ^1^H-NMR (CDCl_3_) δ 12.10 (s, 1H), 8.16 (d, *J* = 8. 6 Hz, 1H), 7.39 (td, *J* = 8.1, 4.7 Hz, 1H), 7.22 (ddd, *J* = 12.7, 7.7, 1.1 Hz, 1H), 7.05 (d, *J* = 10.0 Hz, 1H), 5.67 (d, *J* = 10.0 Hz, 1H), 4.03 (s, 3H), 1.51 (s, 6H); ^13^C-NMR (100 MHz, CDCl_3_) δ 172.1, 158.8, 155.5, 141.3, 129.1, 127.5, 126.2, 122.0, 120.0, 119.1, 115.3, 114.1, 103.2, 74.5, 52.5, 26.6; MS (ESI, *m*/*z*): calcd for C_17_H_16_FO_4_ [M + H]^+^ 303, found 303.

*Methyl 6-hydroxy-2,2,8-trimethyl-2H-benzo[h]chromene-5-carboxylate* (**14b**): Yield: 65%; yellow solid; ^1^H-NMR (CDCl_3_) δ 12.13 (s, 1H), 8.14 (s, 1H), 8.07 (d, *J* = 8. 6 Hz, 1H), 7.39–7.52 (d, *J* = 8. 6 Hz, 1H), 7.10 (d, *J* = 10.0 Hz, 1H), 5.65 (d, *J* = 10.0 Hz, 1H), 4.01 (s, 3H), 2.53 (s, 3H), 1.48 (m, 6H); MS (ESI, *m*/*z*): calcd for C_18_H_19_O_4_ [M + H]^+^ 299, found 299.

*Methyl 6-hydroxy-2,2-dimethyl-8-(trifluoromethyl)-2H-benzo[h]chromene-5-carboxylate* (**14c**): Yield: 66%; yellow solid; ^1^H-NMR (CDCl_3_) δ 12.21 (s, 1H), 8.67 (s, 1H), 8.27 (d, *J* = 8.8 Hz, 1H), 7.75 (dd, *J* = 8.8, 1.7 Hz, 1H), 7.13 (d, *J* = 10.0 Hz, 1H), 5.74 (d, *J* = 10.0 Hz, 1H), 4.04 (s, 3H), 1.50 (s, 6H); MS (ESI, *m*/*z*): calcd for C_18_H_16_F_3_O_4_ [M + H]^+^ 353, found 353.

*Methyl 9-fluoro-6-hydroxy-2,2-dimethyl-2H-benzo[h]chromene-5-carboxylate* (**14d**): Yield = 71%; yellow solid; ^1^H-NMR (CDCl_3_) δ 12.23 (s, 1H), 8.37 (dd, *J* = 9.1, 5.6 Hz, 1H), 7.75 (dd, *J* = 10.5, 2.5 Hz, 1H), 7.18–7.32 (m, 1H), 7.11 (d, *J* = 10.0 Hz, 1H), 5.70 (d, *J* = 10.0 Hz, 1H), 4.02 (s, 3H), 1.48 (s, 6H); MS (ESI, *m*/*z*): calcd for C_17_H_16_FO_4_ [M + H]^+^ 303, found 303. 

### 3.11. General Method I *(*Compounds ***15a**–**d**)*

Various methyl 6-hydroxy-2*H*-benzo[*h*]chromene-5-carboxylates **14a**–**c** (3 mmol), sodium methoxide (2.0 equiv) and an alkyl alcohol (7.0 equiv) were dissolved in anhydrous toluene and added to a microwave vial, which was placed in the microwave cavity, and stirred at 150 °C for 1.5 h. After cooling and being neutralized via addition of 1.0 equivalent of the 6N hydrogen chloride solution, the mixture was concentrated under vacuum. The product was purified by flash chromatography on silica gel using hexane/ethyl acetate (30 to 100% 20 min). Product obtained as a pale yellow oil. Yield 40–59%.

*2-Morpholinoethyl 8-fluoro-6-hydroxy-2,2-dimethyl-2H-benzo[h]chromene-5-carboxylate* (**15a**): Yield: 59%; yellow oil; ^1^H-NMR (CDCl_3_) δ 11.64 (s, 1H), 8.15 (d, *J* = 8.4 Hz, 1H), 7.38 (m, 1H), 7.20 (m, 1H), 7.08 (d, *J* = 10.0 Hz, 1H), 5.66 (d, *J* = 10.0 Hz, 1H), 4.56 (t, *J* = 5.6 Hz, 2H), 3.76 (t, *J* = 4.4 Hz, 4H), 2.79 (t, *J* = 5.6 Hz, 2H), 2.59 (m, 4H), 1.50 (s, 6H); ^13^C-NMR (CDCl_3_) δ 169.8, 158.9, 153.1, 141.3, 129.3, 127.6, 126.1, 121.7, 119.9, 118.8, 114.8, 114.3, 104.9, 74.8, 66.8, 61.6, 56.5, 53.5, 26.7; MS (ESI, *m*/*z*): calcd for C_22_H_25_FO_5_ [M + H]^+^ 402, found 402.

*2-Morpholinoethyl 6-hydroxy-2,2,8-trimethyl-2H-benzo[h]chromene-5-carboxylate* (**15b**): Yield: 41%; yellow oil; ^1^H-NMR (CDCl_3_) δ 8.14 (s, 1H), 8.07 (d, *J* = 8.6 Hz, 1H), 7.48 (dd, *J* = 8.5, 1.8 Hz, 1H), 6.93 (dd, *J* = 10.0, 3.2 Hz, 1H), 5.65 (d, *J* = 10.0 Hz, 1H), 4.87 (m, 2H), 3.97 (m, 4H), 3.68 (m, 2H), 3.53 (m, 2H), 2.99 (m, 2H), 2.54 (s, 3H), 1.47 (s, 6H); MS (ESI, *m*/*z*): calcd for C_23_H_28_NO_5_ [M + H]^+^ 398, found 399.

*2-Morpholinoethyl 6-hydroxy-2,2-dimethyl-8-(trifluoromethyl)-2H-benzo[h]chromene-5-carboxylate* (**15c**): Yield: 45%; yellow oil; ^1^H-NMR (CDCl_3_) δ 11.57 (s, 1H), 8.65 (s, 1H), 8.26 (d, *J* = 8.8 Hz, 1H), 7.73 (dd, *J* = 8.7, 1.6 Hz, 1H), 7.17 (d, *J* = 10.0 Hz, 1H), 5.73 (d, *J* = 10.0 Hz, 1H), 4.58 (t, *J* = 5.6 Hz, 2H), 3.71–3.84 (m, 4H), 2.80 (t, *J* = 5.6 Hz, 2H), 2.53–2.66 (m, 4H), 1.50 (s, 6H); ^13^C-NMR (CDCl_3_) *δ* 170.6, 156.8, 141.7, 130.5, 130.3, 128.4, 125.4, 124.4, 124.1, 123.2, 122.1, 121.9, 114.0, 102.7, 75.1, 63.7, 59.6, 56.3, 52.8, 26.7; MS (ESI, *m*/*z*): calcd for C_23_H_25_F_3_NO_5_ [M + H]^+^ 452, found 452.

*2-Morpholinoethyl 9-fluoro-6-hydroxy-2,2-dimethyl-2H-benzo[h]chromene-5-carboxylate* (**15d**): Yield = 55%; yellow oil; ^1^H-NMR (CDCl_3_) δ 8.37 (dd, *J* = 9.1, 5.6 Hz, 1H), 7.75 (dd, *J* = 10.3, 2.5 Hz, 1H), 7.26 (m, 1H), 6.95 (d, *J* = 10.0 Hz, 1H), 5.71 (d, *J* = 10.0 Hz, 1H), 4.88 (m, 2H), 3.98 (m, 4H), 3.64 (m, 2H), 3.51 (m, 2H), 2.99 (m, 2H), 1.48 (s, 6H); MS (ESI, *m*/*z*): calcd for C_22_H_25_FO_5_ [M + H]^+^ 402, found 402.

### 3.12. Synthesis of ***17a**–**b***

To a benzoic acid (10 mmol) solution in CH_2_Cl_2_ (20 mL), SOCl_2_ (5 mL) was added via syringe. After addition of DMF (3 drops), the reaction mixture was stirred for 5 h at room temperature, and then concentrated to dryness under reduced pressure. The produced acid chloride was dissolved in CH_2_Cl_2_ (30 mL) and cooled under ice bath. Diethylamine (25 mmol) was added dropwise and the resulting colorless suspension was stirred for 3 h at room temperature. After addition of brine (80 mL) and CH_2_Cl_2_ (50 mL), the organic layer was partitioned, washed with brine, dried with MgSO_4_, filtered, and concentrated under reduced pressure to yield the diethylamide which was purified by flash column chromatography to provide the target compound.

*2-Bromo-N,N-diethyl-5-fluorobenzamide* (**17a**): Yield: 97%; colorless oil; ^1^H-NMR (CDCl_3_) *δ* 7.53 (m, 1H), 7.00–6.95 (m, 2H), 3.82 (q, *J* = 6.8 Hz, 1H), 3.33 (q, *J* = 7.2 Hz, 1H), 3.20–3.12 (m, 2H), 1.27 (t, *J* = 6.8 Hz, 3H), 1.09 (t, *J* = 7.2 Hz, 3H); ^13^C-NMR (CDCl_3_) *δ* 167.0, 161.7, 140.3, 134.3, 117.2, 114.7, 113.5, 42.7, 39.0, 13.8, 13.6; MS (ESI, *m*/*z*): calcd for C_11_H_14_BrFNO [M + H]^+^ 274, found 274.

*2-Bromo-N,N-diethyl-4-fluorobenzamide* (**17b**): Yield: 97%; colorless oil; ^1^H-NMR (CDCl_3_) *δ* 6.94 (m, 1H), 6.41–6.29 (m, 4H), 5.74 (s, 3H), 2.53 (q, *J* = 7.5 Hz, 4H), 1.16 (m *J* = 7.5 Hz, 6H).

### 3.13. Synthesis of ***18a**–**b*** from ***17a**–**b***

To a solution of amide **17a**–**b** (9.0 mmol) in dry THF (30 mL), BuLi (1.6 M, 1.3 eq) was added dropwise at −78 °C. The resulting pale yellow solution was stirred for 15 min at the same temperature. DMF (2.5 eq) was added dropwise, and the reaction mixture was stirred for 30 min before slowly warming to ambient temperature over 2 h. The reaction mixture was quenched with a saturated solution of NH_4_Cl (50 mL) and extracted with CH_2_Cl_2_ (3 × 35 mL). The combined organic phase was dried over anhydrous Na_2_SO_4_, filtered, and concentrated under reduced pressure to afford a crude orange oil. Purification of the crude oil by flash chromatography afforded the target products **18a**–**b**.

*N,N-Diethyl-5-fluoro-2-formylbenzamide* (**18a**): Yield: 42%; colorless oil; ^1^H-NMR (CDCl_3_) *δ* 10.37 (s, 1H), 7.62 (dd, *J* = 5.2, 3.2 Hz, 1H), 7.20 (dd, *J* = 8.8, 5.6 Hz, 1H), 7.10 (d, *J* = 7.6 Hz, 1H), 3.60 (q, *J* = 7.2 Hz, 2H), 3.08 (q, *J* = 7.2 Hz, 2H), 1.33 (t, *J* = 7.2 Hz, 3H), 1.03 (t, *J* = 7.2 Hz, 3H); ^13^C-NMR (CDCl_3_) *δ* 186.6, 168.2, 164.7, 139.6, 136.1, 122.9, 120.9, 116.7, 42.6, 38.9, 13.6, 12.2; MS (ESI, *m*/*z*): calcd for C_12_H_15_FNO_2_ [M + H]^+^ 224, found 224.

*N,N-Diethyl-4-fluoro-2-formylbenzamide* (**18b**): Yield: 55%; colorless oil; ^1^H-NMR (CDCl_3_) *δ* 10.02 (s, 1H), 7.64 (m, 1H), 7.37 (m, 2H), 3.62 (q, *J* = 7.1 Hz, 2H), 3.14 (q, *J* = 7.1 Hz, 2H), 1.31 (t, *J* = 7.1 Hz, 3H), 1.07 (t, *J* = 7.1 Hz, 3H); MS (ESI, *m*/*z*): calcd for C_12_H_15_FNO_2_ [M + H]^+^ 224, found 224.

### 3.14. Synthesis of ***19a**–**b*** from ***18a**–**b***

To a solution of the formylarylamide (0.85 g, 4.80 mmol) in CH_2_Cl_2_ (20 mL) at 0–5 °C was added 18-crown-6 (127 mg, 0.48 mmol) and KCN (31 mg, 0.48 mmol). After 10 min, TMSCN (667 mg, 6.7 mmol) was added dropwise and the mixture was stirred for 30 min. The solvent was removed under reduced pressure and AcOH (5 mL) was added to the residue. After stirring for 12 h at room temperature, the reaction mixture was slowly poured into a saturated solution of NaHCO_3_ (100 mL) with stirring. After 30 min (no more gas evolution), organic materials were extracted using ethyl acetate (50 mL), washed with brine, dried with MgSO_4_, filtered, and dried under reduced pressure. Purification of the crude product by flash chromatography afforded the target compounds. 

*5-Fluoro-3-oxo-1,3-dihydroisobenzofuran-1-carbonitrile* (**19a**): Yield: 42%; white gel; ^1^H-NMR (CDCl_3_) δ 7.83 (d, *J* = 7.7 Hz, 1H), 7.76 (m, 1H), 7.5 (m, 1H), 6.17 (s, 1H); ^13^C-NMR (CDCl_3_) *δ* 166.2, 156.8, 134.2, 127.8, 127.3, 122.6, 122.4, 112.5, 63.1; MS (ESI, *m*/*z*): calcd for C_9_H_5_FNO_2_ [M + H]^+^ 178, found 178.

*4-Fluoro-3-oxo-1,3-dihydroisobenzofuran-1-carbonitrile* (**19b**): Yield: 51%; white gel; ^1^H-NMR (CDCl_3_) δ 8.02 (m, 1H), 7.43 (m, 2H), 6.08 (s, 1H); MS (ESI, *m*/*z*): calcd for C_9_H_5_FNO_2_ [M + H]^+^ 178, found 178.4. Biological methods.

## 4. Biological Methods

### 4.1. Materials

RPMI 1640 medium and fetal bovine serum (FBS) were from HyClone-Pierce Co. (Logan, UT, USA), and penicillin-streptomycin and trypsin/EDTA were from WelGENE (Gyeongsan, Korea). 5-Aminosalicylic acid (5-ASA) and DMSO (dimethyl sulfoxide) were purchased from Sigma-Aldrich. 

### 4.2. Cell Cultures

HT-29 human colonic epithelial cells and U937 human pre-monocytic cells were grown in RPMI 1640 medium supplemented with 10% FBS, 100 IU/mL penicillin, and 100 µg/mL streptomycin in a humidified incubator under 5% CO_2_/95% air. 

### 4.3. Cell Adhesion Assay (Monocytic Cells and Epithelial Cells)

U937 monocytic cell adhesion to colonic epithelial cells was evaluated using human U937 pre-monocytic cells, which were prelabeled with 2′,7′-bis(2-carboxyethyl)-5(6)-carboxyfluorescein acetoxymethyl ester (BCECF/AM, 10 μg/mL) for 1 h at 37 °C. HT-29 cells cultured in 24-well plates were pretreated with test compound, 5-ASA, or tofacitinib for 1 h prior to incubation with TNF-α (10 ng/mL) and IL-6 (5 ng/mL) for an additional 3 h. Cells were then co-incubated with BCECF/AM-prelabeled U937 cells (1 × 10^6^ cells/well) for 30 min at 37 °C. Non-adhering U937 cells were removed, and the HT-29 cells and adherent U937 cells were washed twice with PBS. Images were captured and processed with a Nikon ECLIPSE TE2000-U microscope and NIS-Elements F software (Nikon, Tokyo, Japan). For quantitative analysis, other sets of cells were lysed in 0.1% Triton X-100 in Tris (0.1 M), and the fluorescence was measured using a fluorescence-detecting microplate reader (Promega , Madison, WI, USA) at excitation and emission wavelengths of 580 nm. Experiments were performed in triplicate and repeated at least three times independently.

### 4.4. Measurement of SAA Level by ELISA

The level of Serum Amyloid A (SAA) was measured using a mouse SAA ELISA kit according to the manufacturer’s instructions (E-90SAA, Immunology Consultants Laboratory, Inc., Portland, OR, USA). All samples were assayed in duplicate. The concentrations of SAA were determined by comparison to serial dilutions of purified SAA calibrator.

### 4.5. Measurement of Metabolic Stability

Pooled human liver microsomes (150 donor) and dog, rat, mouse liver microsomes were obtained from BD Gentest Co. (Woburn, MA, USA). NADPH regeneration system was obtained from Promega. S-Mephenytoin, 4-hydroxymephenytoin, and 1’-hydroxymidazolam were purchased from Ultrafine Chemical Co. (Manchester, UK). All other chemicals and solvents were of the highest grade available. The metabolic stability assay was performed by incubation of human or selected animal liver microsomes (most often dog, rat and mouse), at 37 °C with a test compound at a final concentration of 1 μM, in the presence of 0.5 mg/mL microsomal protein and NADPH regeneration system, in a total volume of 100 μL of 100 mM phosphate buffer, pH 7.4. The incubation was started by the addition of NADPH regeneration system and terminated with 40 μL of ice-cold acetonitrile at 0 and 30 min. Precipitated proteins were removed by centrifugation at 10,000× *g* for 5 min at 4 °C. Aliquots of the supernatant were injected onto an LC-MS/MS system. Incubations terminated prior to the addition of NADPH regeneration system (time point 0 min) were used as standards, defined as 100%. Percent of the parent compound remaining was calculated by comparing peak area. 

### 4.6. Measurement of Plasma Stability

The plasma stability assay was performed by incubation of human or rat plasma with a test compound in a final concentration of 1 μM for 120 min at 37 °C. The incubation was terminated by addition of 40 μL of ice-cold acetonitrile and vortexing for 5 min. The precipitated proteins were removed by centrifugation at 14,000× *g* for 5 min at 4 °C. Aliquots of the supernatant were injected onto a Shimadzu Nexera XR LC system. Percentages of the parent compound remaining were calculated by comparing peak areas using the Xcalibur software (version 1.6.1, Waltham, MA, USA).

### 4.7. hERG Assay

hERG channel binding assay was performed using Invitrogen’s hERG Fluorescence Polarization Assay kit (PV5365) with Synergy Neo plate reader (BioTek, Winooski, VT, USA). The binding assay was carried out according to the kit manufacturer’s instructions. 

### 4.8. CYP Inhibition Assay

All incubations were performed in duplicate, and the mean values were used for analysis. The assays of phenacetin O-deethylase, tolbutamide 4-hydroxylase, S-mephenytoin 4-hydroxylase, dextromethorphan O-demethylase and midazolam 1′-hydroxylase activities were determined as probe activities for CYP1A2, CYP2C9, CYP2C19, CYP2D6 and CYP3A, respectively, using cocktail incubation and tandem mass spectrometry. Briefly, incubation reaction was performed with 0.25 mg/mL human liver microsomes in a final incubation volume of 100 μL. The incubation medium contained 100 mM phosphate buffer (pH 7.4) with probe substrates. The incubation mixture containing various inhibitors (10 μM) was pre-incubated for 5 min. After pre-incubation, an NADPH regenerating system was added. After incubation at 37 °C for 15 min, the reaction was stopped by placing the incubation tubes on ice and adding 40 μL of ice-cold acetonitrile. The incubation mixtures were then centrifuged at 10,000× *g* for 5 min at 4 °C. Aliquots of the supernatant were injected onto an LC-MS/MS system. The CYP-mediated activities in the presence of inhibitors were expressed as percentages of the corresponding control values.

### 4.9. Determination of Water Solubility at 10 mM

Water solubility (>10 mM) was determined using the following procedure at room temperature. The test compound was added to a vial containing sodium phosphate buffer, 0.1 M, pH 7.4 (1 mL) to make a 10 mM mixture, and the mixture was equilibrated during 10 min of sonication and then visually checked immediately and after 24 h for any undissolved parts or precipitation of the sample. If no precipitation was shown after 24 h, the aqueous solution was filtered (Syringe filter; PTFE; pore size: 0.45 μm; 25 mm DI) and subjected to LC-MS to confirm the reagent. 

## 5. Conclusions

To improve metabolic stability, plasma stability, and water solubility, we synthesized various mollugin derivatives and assayed their inhibitory activity against U937 monocyte cell adhesion to HT-29 colonic epithelial cells in both TNF-α- and IL-6-induced models of colon inflammation at a 10 μM concentration. The water solubility and plasma stability were improved by replacing the methyl ester of mollugin with 2-(4-morpholinyl)-ethyl, and the metabolic stability was improved by substitution of F- or CF_3_- on the aromatic ring of mollugin. Compound **15c** has good water solubility, plasma stability, and metabolic stability and has greater inhibitory activity than mesalazine or mollugin in the TNF-α- or the IL-6-induced colonic epithelial cell adhesion assay. The results suggest that **15c** is a potential anti-inflammatory agent.

## 6. Patents

Part of this work was disclosed in a patent application: Kongkae Taeho Kongbo 2017, KR 2017122970 A 20171107).

## Figures and Tables

**Table 1:** % inhibition, metabolic stability, plasma stability, hERG binding of active compounds.

	% Inhibition (at 10 uM)		Metabolic Stability (% Remain, 30 min)				Plasma Stability (% Remain, 2 h)		hERG IC_50_ (μM)
	TNF-α	IL-6	Human	Dog	Rat	Mouse	Human	Rat	
**2** (5-ASA)	28.8 ± 7.7 (at 20 mM)	32.6 ± 7.3 (at 20 mM)							
**1** (mollugin)	37.8 ± 5.7	40.8 ± 6.2	33	33	19	40	92	16	>10
**6a**	38.0 ± 4.9	35.5 ± 4.3	6	2	2	3			
**6b**	39.6 ± 2.9	34.8 ± 4.1	12	9	7	8			
**6c**	39.7 ± 5.7	12.4 ± 2.1							
**6d**	46.3 ± 6.2	26.5 ± 5.0	83	45	20	10	97	97	>10
**6e**	40.1 ± 3.5	26.6 ± 5.1	36	42	16	24	100	94	>10
**6f**	48.3 ± 6.2	38.2 ± 5.1	58	12	13	11	100	99	>10
**6g**	54.8 ± 5.2	38.9 ± 5.0	78	36	23	14	100	100	>10
**6h**	42.2 ± 6.4	18.2 ± 3.1	76	11	23	9	79	89	3
**6i**	58.5 ± 6.4	27.6 ± 4.2							5
**6j**	43.1 ± 6.2	33.6 ± 5.9							
**6k**	42.6 ± 4.2	39.1 ± 4.7	10	6	2	2			
**6l**	45.4 ± 4.7	12.2 ± 4.4	13	1	1	2			
**6m**	38.4 ± 5.7	25.8 ± 3.4							
**6n**	37.4 ± 3.3	34.2 ± 7.9							
**6o**	46.2 ± 5.4	9.4 ± 2.1							

**Table 2:** % inhibition, metabolic stability, plasma stability, hERG binding of active compounds.

#	% Inhibition (at 10 uM)		Metabolic Stability (% Remain, 30 min)				Plasma Stability (% Remain, 2 h)	
	TNF-α	IL-6	Human	Dog	Rat	Mouse	Human	Rat
**14a**	36.1 ± 7.6	59.4 ± 7.4	52	52	78	82	39.5	2
**14b**	29.8 ± 7.2	23.4 ± 2.5	88	4	51	19	100	1
**14c**	63.2 ± 10.8	39.3 ± 5.6	100	22	90	70	97	10
**14d**	24.9 ± 8.1	28.0 ± 5.6						
**15a**	36.6 ± 9.4	61.1 ± 10.9	78	10	9	10	100	91
**15b**	24.9 ± 3.5	54.3 ± 6.5	41	57	9	18	100	55
**15c**	46.0 ± 7.6	50.3 ± 5.7	84	86	52	45	100	51
**15d**	47.0 ± 8.9	75.6 ± 11.5	48	30	8	11	94	81

**Table 3:** CYP inhibition of selected compounds.

#	CYP Inhibition (% of Control)				
	1A2	2C9	2C19	2D6	3A4
Mollugin	41	93	68	99	98
**6d**	58	99	68	75	91
**15c**	57	97	93	92	90
